# The Effect of Using Smart Glasses Integrated Ultrasonography in Radial Artery Catheterization: A Prospective Randomized Trial

**DOI:** 10.4274/TJAR.2025.252052

**Published:** 2025-12-22

**Authors:** Merve Gözen, Bengi Şafak, Ayşegül Güven, Onat Bermede

**Affiliations:** 1 Ankara University Faculty of Medicine Department of Anesthesiology and Intensive Care, Ankara, Türkiye

**Keywords:** Catheterization, radial artery, smart glasses, ultrasonography

## Abstract

**Objective:**

The use of ultrasonography (USG) in arterial catheterization, in which the comfort of the practitioners and hand-eye coordination are very important, is frequently needed by anesthesiologists in daily practice. We aimed to investigate whether radial artery catheterization with smart glasses-integrated USG would increase success and satisfaction.

**Methods:**

One hundred twenty patients who were >18 years old and would have undergone elective surgery with an indication for radial artery catheterization between August and December 2022 were included in this prospective randomized study. Patients who underwent catheterization in the last month and had contraindications were excluded. In the Standard USG Group, catheterizations were performed with standard USG, and in the Smart Glass Group, with smart glasses-integrated USG. Two anesthetists, a junior practitioner with experience with 20-50 catheterizations and a senior practitioner with experience with over 50 catheterizations, performed the catheterizations. The subcutaneous distance, radial artery depth, and diameter in short axis, catheterization time, and ergonomic satisfaction were recorded.

**Results:**

Sixty patients in standard USG group and 59 patients in Smart Glass Group, with similar demographics, were included in statistical analysis. The mean first catheterization time by junior practitioners, with smart glasses integrated USG, was shorter than standard USG (49.07±29.91 sec vs. 99.73±75.18 sec, *P* <0.001). The junior practitioner was more satisfied with smart glasses-integrated USG. There was no significant difference between groups in terms of interventions made by the senior practitioner.

**Conclusion:**

Radial artery catheterization with smart glasses integrated USG shortens catheterization time, and increases satisfaction by increasing the comfort of USG use for junior practitioners.

**Main Points**

• The use of ultrasonography (USG) in radial artery catheterization significantly increases successful catheterization and minimizes complications.

• Smart glasses allow the practitioner to simultaneously view the screen and the procedure area.

• The radial artery catheterization with smart glasses integrated USG increases the satisfaction of the junior practitioner by shortening the catheterization time.

## Introduction

In daily practice, anesthesiologists commonly perform arterial catheterization for continuous blood pressure monitoring, cardiac output assessment, and blood gas sampling. Because of its proximity to the skin, the radial artery is preferred. Nonetheless, in the presence of obesity, hypotension, or tachycardia, palpation of the radial artery may be difficult.^1^ Thus, complications such as hematoma, thrombosis, or mechanical injury may arise during the procedure.^2^ Compared to the traditional landmark technique, ultrasonography (USG) guidance significantly increases successful catheterization and minimizes complications.^3^ In addition to USG and anatomical knowledge, coordination skills between hand, eye, procedure area, and screen are necessary for USG-guided vascular access.^4^ Extra head and eye movements lengthen the procedure, destabilize the probe position, and result in the loss of the target image and an improper change in needle direction.^5^ Prolonged procedure time and repetitive movements aggravate the practitioner’s musculoskeletal fatigue and affect success.^6^ Smart glasses project the USG image in real-time directly in front of the practitioner’s eyes, allowing the practitioner to simultaneously view the screen and the procedure area.^5^ This innovative technology is attractive for invasive procedures because it allows the practitioner to access various data without touching the device.^7^

The primary aim of this study was to evaluate the success rate and anesthesiologist’s satisfaction of two practitioners with different levels of experience in radial artery catheterization with smart glasses-integrated USG. The secondary aim was to evaluate the cannulation time and complications between groups.

## Methods

This prospective randomized study was conducted between August and December 2022 in Ankara University Hospital after the approval of the Ankara University Medical Faculty Ethics Committee (approval no: I08-498-22, date: 15.09.2022). The study was registered at ClinicalTrials.gov (NCT06271499) and conducted following the principles of the Helsinki Declaration. Patients over 18 years of age who underwent elective surgery with an indication for radial artery catheterization were included. Patients who underwent radial artery catheterization in the last month, and with any contraindication, negative Allen test, and peripheral vascular disease were excluded. Written informed consent was obtained during the preoperative examination.

**Figure 1:**
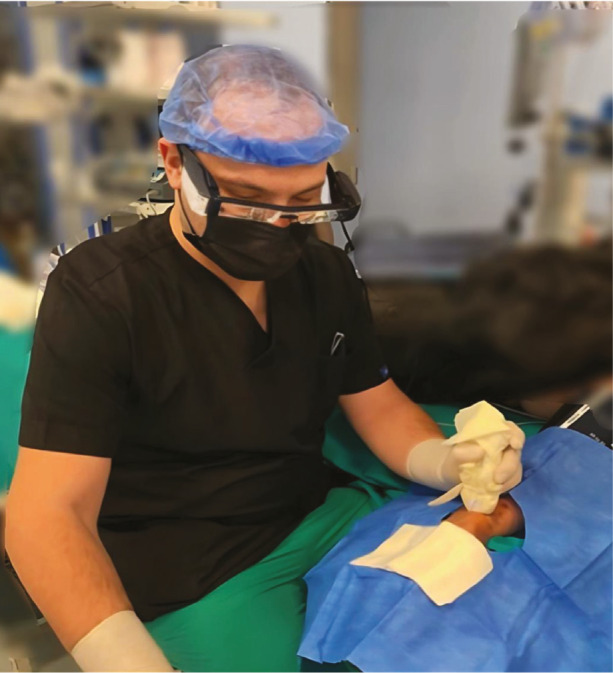
Radial artery catheterization with smart glasses integrated USG. USG, ultrasonography.

Patients were randomly divided into two groups utilizing the sealed envelope method. In the Standard USG Group, patients underwent radial artery catheterization with a wireless USG probe (C10RL 7.5/10 MHz linear probe, Konted, China) connected to a tablet. In Smart Glass Group, the ultrasound image, obtained with the same wireless USG probe, was first transferred to a tablet via Wi-Fi, and then the image was transferred to the smart glasses (Moverio BT-40 model smart glasses, Epson, Korea) via a cable. Thus, with smart glasses, the practitioner was able to visualize both the catheterization site and the ultrasound image simultaneously while performing the catheterization. In Figure 1, the practitioner is using smart glasses during radial artery catheterization. Although the ultrasound image projected on the smart glasses display is not visible in the photo, it was clearly viewable in real-time by the operator during the procedure. The catheterizations were performed by two anesthetists with different experiences: a junior practitioner with experience in 20-50 radial artery catheterizations, and a senior practitioner with experience in over 50 radial artery catheterizations. Since practitioners had no experience with smart glasses before the study, they performed five catheterizations on a model with smart glasses integrated USG.

After general anesthesia induction, the modified Allen test was performed on the non-dominant hand to determine the catheterization side. The wrist was positioned at a 45° angle with support. After skin asepsis, the radial artery was visualized 2-3 centimeters proximal to the wrist in the short axis, and the subcutaneous distance, radial artery depth, and diameter were recorded. The radial artery puncture was performed with step-by-step monitoring of the needle tip in the short axis. Following the puncture, the radial artery was catheterized with a 3 French, 8 cm catheter (VYGON, Arterial Leadercath, France). When the artery waveform was being monitored, the catheterization was considered successful. The time between the puncture and the appearance of the artery waveform was recorded as catheterization time. If the puncture could not be performed, the catheterization was considered unsuccessful. The radial artery was re-imaged after catheterization to assess for any complications, and measurements were recorded in the short axis. In Standard USG Group, catheterizations were performed with the wireless USG probe, and in Smart Glass Group, all catheterizations were performed with the smart glasses-integrated USG probe. The ergonomic satisfaction of the practitioner was evaluated with a 5-point Likert scale (1: Very dissatisfied, 2: Dissatisfied, 3: Undecided, 4: Satisfied, 5: Very satisfied).

### Statistical Analysis

A prior power analysis was conducted using G*Power software (version 3.1.9.2) to estimate the minimum required sample size. The analysis was based on a medium effect size (Cohen’s d=0.5), a significance level (α) of 0.05, a desired statistical power of 80%, and a two-tailed independent samples t-test. The calculation indicated that at least 54 participants per group would be required to detect a statistically significant difference between the groups. The assumed effect size reflects a clinically meaningful difference and corresponds to the standardized difference between group means relative to the pooled standard deviation. Since group means and standard deviations are reported in the results section, the effect size can be inferred. A two-tailed test was used as it is standard practice unless a specific directional hypothesis is being tested.

Quantitative variables were described by mean and standard deviation, and qualitative variables were described by the number of patients (percentage). To determine whether there was a difference between the categories of the qualitative variable and two categories of the quantitative variable, the Student’s t-test was used if the normal distribution assumptions were met, and the Mann-Whitney U test was used otherwise. The relationship between two qualitative variables was analyzed with Chi-square and Fisher’s exact tests. Since the assumptions of normal distribution were not met, Spearman’s correlation test was used to examine the relationship between two quantitative variables. The program SPSS 11.5 was used to analyze the data. *P* <0.05 was defined as the statistical significance level.

## Results

**Figure 2:**
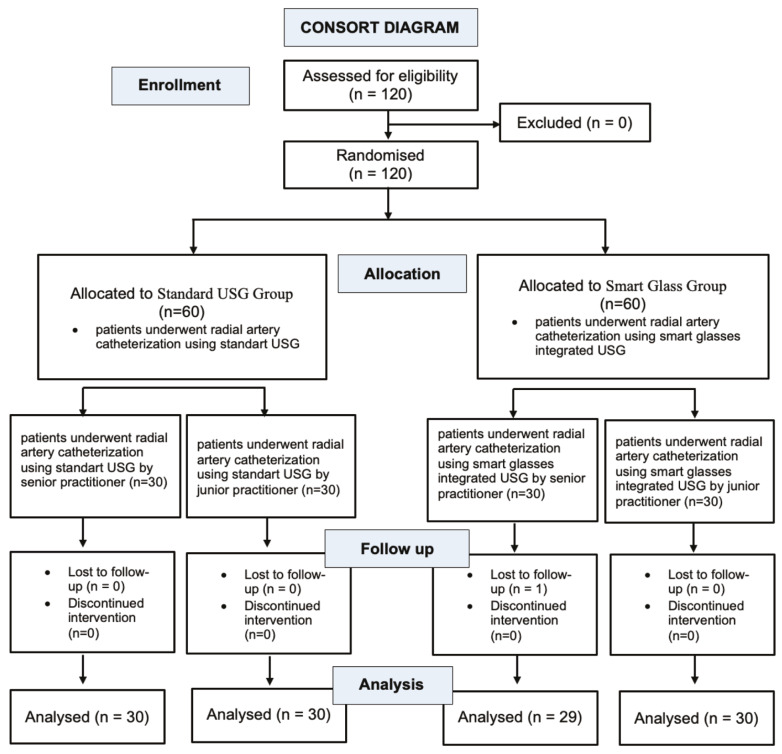
CONSORT flow diagram for the study.

One hundred twenty patients were included to account for the possibility of data loss. The statistical analysis included 60 patients in the Standard USG Group, and 59 patients in the Smart Glass Group (Figure 2). Between the groups studied, demographics were comparable-except for age. In the standard USG group, 7 first catheterizations were unsuccessful, and in the Smart Glass Group, 2 first catheterizations were unsuccessful, and a second catheterization was needed (Table 1). One patient in standard USG group, and one in Smart Glass Group, had a hematoma. During the surgery, the catheter was dysfunctional in one patient of the Standard USG Group and in one patient of the Smart Glass Group.

The junior practitioner was successful on 26 of the first 30 catheterizations with standard USG and 29 of the first 30 catheterizations with smart-glasses-integrated USG. The first catheterization time by a junior practitioner was significantly longer in the Standard USG Group than in the Smart Glass Group. The senior practitioner was successful in 27 of 30 catheterizations with standard USG and 28 of 29 with smart-glasses-integrated USG. The first catheterization time by a senior practitioner was not significantly different between groups. The junior practitioner’s satisfaction levels varied significantly between groups. However, the senior practitioner’s satisfaction level revealed no significant difference between groups (Table 2).

In standard USG group, the junior practitioner’s first catheterization time was longer than the senior practitioner’s (99.73±75.18 sec and 59.13±41.98 sec, respectively, *P*=0.006); however, in Smart Glass Group, there was no significant difference (49.07±29.91 sec and 44.66±32.24 sec, respectively, *P*=0.426). The satisfaction levels of the senior practitioner were significantly higher than that of the junior practitioner in Standard USG Group(3.40±1.04 and 2.60±1.07, respectively, *P*=0.008); however, in Smart Glass Group, there was no significant difference (3.76±0.95 and 3.67±0.96, respectively, *P*=0,665).

In the Standard USG Group, the mean catheterization time with a satisfaction level of 1-2-3 was 100.21±69.21 seconds, and with a satisfaction level of 4-5 was 40.86±21.96 seconds (*P* <0.001). In Smart Glass Group, the mean catheterization time with a satisfaction level of 1-2-3 was 69.64±37.99 seconds, and with a satisfaction level of 4-5 was 33.38±13.83 seconds (*P* <0.001). There was a moderate negative correlation between the catheterization time and anesthesiologist satisfaction in the Standard USG Group (r =-0.698 and *P* <0.001; r =-0.742 and *P *<0.001), and a strong negative correlation in the Smart Glass Group (r =-0.632 and *P *<0.001; r =-0.867 and *P *<0.001).

## Discussion

This study shows that radial artery catheterization by the junior practitioner with smart glasses integrated USG does not affect the success of catheterization; however, it shortens the catheterization time. The decreased catheterization time as a result of improved hand-eye coordination with smart glasses integrated USG boosts the satisfaction rate in comparison to standard USG.

Currently, USG is utilized in daily anesthesia practice. The real-time image obtained by USG during vascular interventions improves the success rate by allowing the anatomy to be understood and the needle tip to be tracked.^7^ Catheter insertion in small vessels can be tricky for even experienced practitioners. USG-guided radial artery catheterization had a higher success rate than the palpation technique in pediatric patients.^8^ A study by Ganesh et al.^9^ comparing USG-guided radial artery catheterization to palpation technique in pediatric patients showed that USG-guided catheterization did not improve the success rate. The success rate was 13.8% in palpation technique and 13.9% in USG-guided catheterization. These low success rates may be attributed to inexperienced clinicians working with the pediatric population. However, in this study, in cases where the practitioner failed with the palpation technique, the more experienced practitioner performed catheterization with USG guidance.^9^

Numerous factors influence the success rates of USG-guided procedures. In the study by Jang et al.^10^ evaluating radial artery catheterization in pediatric patients, the first catheterization success rate was greater in the smart glasses-integrated USG group, than in the USG-guided group. Our population consists of adults with greater radial artery diameters than the paediatric population, which may explain why there was no difference in the first catheterization success rate between the groups. In a study by Kim et al.,^11^ radial artery catheterization in the long-axis was compared between smart glasses integrated USG with laser guidance and smart glasses integrated USG alone. The results showed that first catheterization success, first-pass success, and successful artery catheterization without redirecting the needle were greater with laser guidance.^11^ The practitioner’s experience can be the most significant factor influencing the success rates of USG-guided procedures. A study by Stolz et al.^12^ showed that the success rate of USG-guided peripheral vascular access by learners increases as the number of attempts rises. Since it enhances hand-eye coordination, catheterization using smart glasses integrated USG is more effective for junior practitioners than it is for senior practitioners.^13^ In our study, there was no significant difference in the first catheterization success rate; this outcome can be explained by practitioners completing the learning curves. The relatively high first catheterization success rate observed in our study, especially for the junior practitioner, may be attributed to several factors. A standardized protocol and training were provided prior to the study, which likely enhanced the junior practitioner’s performance. Moreover, patient selection and preparation might have contributed to favorable conditions for catheterization. These factors may have collectively contributed to higher success rates compared to those reported in the literature.

One of the most significant drawbacks of USG-guided catheterizations is that the screen and intervention area are in different locations. This problem impairs hand-eye coordination by causing unwarranted head-neck movements. In a simulation study comparing USG-guided peripheral venous access with and without smart glasses, smart glasses were found to reduce the catheterization time.^14^ With smart glasses integrated ultrasound guidance, the real-time image is kept in line of sight.^15^ Thus, unwanted movements, which can lead to changes in the depth or direction of the needle, diminish. In our study, the junior practitioner completed catheterization faster with smart glasses-integrated USG.

A potential technical limitation of using smart glasses is the risk of connection latency between the ultrasound device and the display. In our study, however, the wireless transmission was stable throughout the procedures, and no noticeable delay was observed that interfered with real-time visualization or catheterization performance. Nevertheless, it is important to note that latency may vary depending on the specific device used and network conditions in other clinical settings, which could potentially affect the generalizability of these results.

Radial artery catheterization with smart glasses integrated USG increases the practitioner’s satisfaction due to the physical comfort, increased success rate, and a reduced catheterization time. Reducing repetitive movements provides practitioners with ergonomic benefits.^10^ In our study, the junior practitioner’s satisfaction was higher with smart glasses integrated USG-guidance. This outcome may be due to the fact that the junior practitioners frequently switch their attention between the USG screen and the intervention area. The use of smart glasses integrated with USG can boost practitioners’ satisfaction by removing distractions. In addition to smart glasses, laser guidance could allow visualization of the needle tip in the sagittal plane in the long-axis view.^11^ The simple and understandable inclusion of swiftly evolving technology in our daily practice can make our lives easier, increasing the technology’s applicability.

Vascular procedures have risks. Even with standard USG guidance, high complication rates might be observed as small-diameter blood vessels are susceptible to injury. In a study by Jang et al.^10^ evaluating radial artery catheterization in pediatric patients, the complication rate was 5.2% in the smart glasses integrated USG group and 29.3% in the standard USG group. Subcutaneous nitroglycerin injection in pediatric patients has been shown to reduce the complication rate from 32.1% to 3.5% in USG-guided catheterization.^16^ A hematoma was observed in one patient in the Standard USG Group, and one patient in the Smart Glass Group, during our study. This may be attributed to our small sample group and the fact that pediatric procedures are more intricate making complications more prevalent.

### Study Limitations

Our study has some limitations. One important limitation of this study is the involvement of only two anaesthesiologists-one junior and one senior practitioner. The limited number of participants may not adequately represent the broader population of anaesthesiologists, potentially introducing practitioner selection bias. The outcomes observed could be influenced by individual skill differences rather than reflecting generalizable trends. Future studies should aim to include a larger and more diverse group of practitioners to minimize potential bias and enhance the external validity of the results. Second, a potential limitation is the influence of the learning curve effect, particularly for the junior practitioner. As the study progressed, the junior practitioner might have gained experience and improved performance, which could have impacted the outcomes. Although procedural standardization was used to minimize variability, the learning effect could not be entirely avoided and should be considered when interpreting the results. Third, this study was conducted in a single center with a specific population. As such, findings may not be completely generalizable; multicenter studies are needed to validate these results across wider populations. Another limitation is that the head and neck movements of practitioners were not evaluated. It may be beneficial to analyze the practitioner‘s physical comfort objectively. Lastly, there was no documented imaging time for the radial artery. Using smart-glasses-integrated USG may affect the time between the beginning of a procedure and when it is acquiring the correct image.

## Conclusion

Technical and ergonomic improvements enhance our daily clinical practice. The radial artery catheterization with smart glasses integrated USG keeps the real-time image within the practitioner’s line of sight, and increases the satisfaction of the junior user by shortening the catheterization time. Consequently, in a continuously changing world, new studies are required so that technology can play an active role in clinical practice.

## Ethics

**Ethics Committee Approval:** This prospective randomized study was conducted between August and December 2022 in Ankara University Hospital after the approval of the Ankara University Medical Faculty Ethics Committee (approval no: I08-498-22, date: 15.09.2022).

**Informed Consent:** Written informed consent was obtained from all the participants.

## Figures and Tables

**Table 1. Patients’ Demographics and Measured Parameters:** 

-		**Group**		***P *value**
		**Standard USG group (n:60)**	**Smart Glass Group (n:59)**	
Gender, n (%)	Female	20 (33.3)	22 (37.3)	0.652
	Male	40 (66.7)	37 (62.7)	
Age​ (year)	Mean ± SD	60.97±11.30	65.85±12.29	0.026*
BMI​	Mean ± SD	27.93±5.19	26.63±5.03	0.169
Hypertension, n (%)	Yes	35 (58.3)	36 (61.0)	0.765
	No	25 (41.7)	23 (39.0)	
Diabetes, n (%)	Yes	21 (35.0)	23 (39.0)	0.653
	No	39 (65.0)	36 (61.0)	
Hyperlipidemia, n (%)	Yes	25 (41.7)	25 (42.4)	0.938
	No	35 (58.3)	34 (57.6)	
Subcutaneous distance (mm)	Mean ± SD	2.9±1.0	2.8±0.8	0.549
Pre-catheterization depth (cm)	Mean ± SD	2.4±0.6	2.4±0.5	0.814
Pre-catheterization diameter (cm)	Mean ± SD	2.6±0.7	2.8±0.7	0.637
Post-catheterization depth (cm)	Mean ± SD	2.4±0.6	2.4±0.6	0.858
Post-catheterization diameter (cm)	Mean ± SD	2.6±0.7	2.5±0.7	0.485
First catheterization, n (%)	Successful	53 (88.3)	57 (96.6)	0.163
	Unsuccessful	7 (11.7)	2 (3.4)	
First catheterization time (sec)	Mean ± SD	79.43±63.74	46.90±30.89	<0.001*
Second catheterization time (sec)	Mean ± SD	74.50±44.20	74.50±38.89	1.000

BMI, body mass index; cm, centimeter; sec, seconds SD, standard deviation; *P <0.05 is taken as statistically significant.

**Table 2. Comparison of Veriables Between Groups:** 

**Experience**	**Variables**		**Group**		***P* value**
			**Standard USG group**	**Smart Glass Group**	
**The junior practitioner**	First catheterization, n (%)	Successful	26 (86.7)	29 (96.7)	0.353
		Unsuccessful	4 (13.3)	1 (3.3)	
	First catheterization time (sec)	Mean ± SD	99.7±75.9	49.1±29.9	<0.001*
	Second catheterization time (sec)	Mean ± SD	99.3±23.2	102.00±-	1.000
	Satisfaction Level, n (%)	1	7 (23.3)	0 (0.0)	0.001*
		2	4 (13.3)	4 (13.3)	
		3	13 (43.4)	8 (26.7)	
		4	6 (20.0)	12 (40.0)	
		5	0 (0.0)	6 (20.0)	
	Satisfaction level	Mean ± SD	2.6±1.05	3.67±0.94	<0.001*
**The senior practitioner**	First catheterization. n (%)	Successful	27 (90.0)	28 (96.6)	0.612
		Unsuccessful	3 (10.0)	1 (3.4)	
	First catheterization time (sec)	Mean ± SD	59.1±42.0	44.7±32.2	0.106
	Second catheterization time (sec)	Mean ± SD	25.0±28.3	47.00±-	0.667
	Satisfaction level, n (%)	1	1 (3.3)	1 (3.4)	0.527
		2	5 (16.7)	1 (3.4)	
		3	9 (30.0)	8 (27.6)	
		4	11 (36.7)	13 (44.9)	
		5	4 (13.3)	6 (20.7)	
	Satisfaction level	Mean ± SD	3.40±1.02	3.76±0.93	0.172

SD, standard deviation; sec, seconds; *P <0.05 is taken as statistically significant.
